# Retracted: Fractalkine Attenuates Microglial Cell Activation Induced by Prenatal Stress

**DOI:** 10.1155/2018/6924505

**Published:** 2018-05-22

**Authors:** Neural Plasticity

Neural Plasticity has retracted the article titled “Fractalkine Attenuates Microglial Cell Activation Induced by Prenatal Stress” [1]. Figures 5 and 6 show signs of figure duplication. In Figure 5, β-actin lanes 1–3 and lanes 4–6 appear to be the same but rotated. In Figure 6, CX3CR1 lanes 1-2 and β-actin lanes 7-8 appear to be the same and CX3CR1 lanes 2 and 7 appear to be the same. The authors provided the underlying blots for Figures 1, 5, and 6, but these blots do not all match the corresponding published images and there was undeclared splicing.

The first author would like to apologize to the other authors, to the readers and editors of Neural Plasticity for these graphical errors. All authors agree to the retraction.
